# 998. Forward and Reverse Translational Approaches to Predict Efficacy of the Neutralizing Respiratory Syncytial Virus (RSV) Antibody MK-1654

**DOI:** 10.1093/ofid/ofab466.1192

**Published:** 2021-12-04

**Authors:** Brian M Maas, Jos Lommerse, Nele Plock, Radha Railkar, S Y Amy Cheung, Luzelena Caro, Jingxian Chen, Wen Liu, Ying Zhang, Qinlei Huang, Wei Gao, Li Qin, Jie Meng, Han Witjes, Emilie Schindler, Benjamin Guiastrennec, Francesco Bellanti, Daniel Spellman, Brad Roadcap, Mariya Kalinova, Juin Fok-Seang, Andrew P Catchpole, Amy Espeseth, S Aubrey Stoch, Eseng Lai, Kalpit A Vora, Antonios O Aliprantis, Jeffrey R Sachs

**Affiliations:** 1 Merck & Co., Inc., Kenilworth, New Jersey; 2 Certara, Princeton, New Jersey; 3 hVivo Services Ltd., London, England, United Kingdom; 4 Merck.& Co., Inc., West Point, PA

## Abstract

**Background:**

MK-1654 is a respiratory syncytial virus (RSV) F glycoprotein neutralizing monoclonal antibody (mAb) with an extended half-life in late development to prevent RSV infection in infants. Neutralizing mAbs, like MK-1654, have great potential for prophylaxis against viral infection. However, well-validated approaches for clinical dose and efficacy predictions are lacking.

**Methods:**

Summary-level literature data from RSV prevention studies were used in a model-based meta-analysis (MBMA) to describe the relationship between RSV incidence rates and serum neutralizing antibody (SNA) titer. The model was validated using viral challenge experiments in cotton rats and phase 3 RSV-A efficacy results in infants for an anti-RSV F mAb, REGN-2222. A phase 2b human RSV challenge study (HCS) in adults was also conducted with MK-1654. Participants (N=70) received 100, 200, 300, or 900 mg of MK-1564 or placebo and were challenged intranasally with RSV 29 days later. RSV viral load and symptomatic infection were monitored. Data from the HCS were compared to model predictions. The MBMA was used to predict efficacy of MK-1654 in a virtual population of pre- and full- term infants.

**Results:**

The relationship between SNA titer and RSV incidence rate defined using the viral load data from the cotton rat approximated the relationship identified for infants from the clinical MBMA. The MBMA was quantitatively consistent with the phase 3 efficacy results against RSV A for REGN-2222. In the HCS, RSV nasal viral load measured by RT-qPCR and quantitative culture as well as symptomatic infections were decreased in MK-1654 recipients compared to placebo. Incidence rates of RSV infection in the HCS were also consistent with MBMA predictions. The model-based clinical trial simulations for MK-1654 indicated a high probability of substantial efficacy against RSV-associated medically attended lower respiratory tract infection ( >75% for 5 months) for doses ≥75 mg.

**Conclusion:**

Our MBMA successfully quantified the relationship between RSV SNA and clinically relevant endpoints, including lower respiratory tract infection in infants. MBMA-based efficacy predictions support continued development of the MK-1654 antibody for the prevention of RSV in infants.

**Disclosures:**

**Brian M. Maas, PharmD**, **Merck & Co., Inc.** (Employee, Shareholder) **Jos Lommerse, PhD**, **Certara** (Employee, Shareholder)**Merck & Co., Inc.** (Independent Contractor) **Nele Plock, PhD**, **Certara** (Employee, Shareholder)**Merck & Co., Inc.** (Independent Contractor) **Radha Railkar, PhD**, **Merck & Co., Inc.** (Employee, Shareholder) **S. Y. Amy Cheung, PhD**, **Certara** (Employee, Shareholder) **Luzelena Caro, PhD**, **Merck & Co., Inc.** (Employee, Shareholder) **Jingxian Chen, PhD**, **Merck & Co., Inc.** (Employee, Shareholder) **Wen Liu, MPH**, **Merck & Co., Inc.** (Employee, Shareholder) **Ying Zhang, PhD**, **Merck & Co., Inc.** (Employee, Shareholder) **Qinlei Huang, MS**, **Merck & Co., Inc.** (Employee, Shareholder) **Wei Gao, PhD**, **Merck & Co., Inc.** (Employee, Shareholder) **Li Qin, PhD**, **Certara** (Employee, Shareholder)**Merck & Co., Inc.** (Independent Contractor) **Jie Meng, MSc**, **Certara** (Employee, Shareholder)**Merck & Co., Inc.** (Independent Contractor) **Han Witjes, PhD**, **Certara** (Employee, Shareholder)**Merck & Co., Inc.** (Independent Contractor) **Emilie Schindler, PhD**, **Certara** (Employee, Shareholder)**Merck & Co., Inc.** (Independent Contractor) **Benjamin Guiastrennec, PharmD, PhD**, **Certara** (Employee, Shareholder)**Merck & Co., Inc.** (Independent Contractor) **Francesco Bellanti, PhD**, **Certara** (Employee, Shareholder)**Merck & Co., Inc.** (Independent Contractor) **Daniel Spellman, PhD**, **Merck & Co., Inc.** (Employee, Shareholder) **Brad Roadcap, MS**, **Merck & Co., Inc.** (Employee, Shareholder) **Amy Espeseth, PhD**, **Merck & Co., Inc.** (Employee, Shareholder) **S. Aubrey Stoch, MD**, **Merck & Co., Inc.** (Employee, Shareholder) **Eseng Lai, MD, PhD**, **Merck & Co., Inc.** (Employee, Shareholder) **Kalpit A. Vora, PhD**, **Merck & Co., Inc.** (Employee, Shareholder) **Antonios O. Aliprantis, MD, PhD**, **Merck & Co., Inc.** (Employee, Shareholder) **Jeffrey R. Sachs, PhD**, **Merck & Co., Inc.** (Employee, Shareholder)

